# Screening potential biomarkers of cholangiocarcinoma based on gene chip meta-analysis and small-sample experimental research

**DOI:** 10.3389/fonc.2022.1001400

**Published:** 2022-10-10

**Authors:** Hengyan Shen, Xinyu Bai, Jie Liu, Ping Liu, Tao Zhang

**Affiliations:** ^1^ Department of Laboratory Medicine, Affiliated Hospital of Zunyi Medical University, Zunyi, China; ^2^ Key Laboratory of Basic Pharmacology of Ministry of Education and Joint International Research Laboratory of Ethnomedicine of Ministry of Education, Zunyi Medical University, Zunyi, China

**Keywords:** cholangiocarcinoma, bioinformatics, meta-analysis, differentially expressed genes, biomarkers

## Abstract

Cholangiocarcinoma (CCA) is a rare malignant tumor associated with poor prognosis. This study aimed to identify CCA biomarkers by investigating differentially expressed genes (DEGs) between CCA patients and healthy subjects obtained from the Gene Expression Omnibus database. Bioinformatics tools, including the Illumina BaseSpace Correlation Engine (BSCE) and Gene Expression Profiling Interactive Analysis (GEPIA), were used. The initial DEGs from GSE26566, GSE31370, and GSE77984 were analyzed using GEO2R and Venn, and protein–protein interaction networks were constructed using STRING. The BSCE was applied to assess curated CCA studies to select additional DEGs and them DEGs across the 10 biosets, which was supported by findings in the literature. The final 18 DEGs with clinical significance for CCA were further verified using GEPIA. These included *CEACAM6*, *EPCAM*, *LAMC2*, *MMP11*, *KRT7*, *KRT17*, *KRT19*, *SFN*, and *SOX9*, which were upregulated, and *ADH1A*, *ALDOB*, *AOX1*, *CTH*, *FGA*, *FGB*, *FGG*, *GSTA1*, and *OTC*, which were downregulated in CCA patients. Among these 18 genes, 56 groups of genes (two in each group) were significantly related, and none were independently and differentially expressed. The hub genes *FGA*, *OTC*, *CTH*, and *MMP11*, which were most correlated with the 18 DEGs, were screened using STRING. The significantly low expression of *FGA*, *OTC*, and *CTH* and significantly high expression of *MMP11* were verified by immunohistochemical analysis. Overall, four CCA biomarkers were identified that might regulate the occurrence and development of this disease and affect the patient survival rate, and they have the potential to become diagnostic and therapeutic targets for patients with CCA.

## Introduction

Cholangiocarcinoma (CCA) is a rare malignant tumor associated with poor prognosis (1), accounting for 2% of all malignant tumors. In recent decades, the incidence of this disease in Asia and other countries has increased significantly (2). CCA is divided into intrahepatic (iCCA), perihepatic, and distal hepatic cholangiocarcinoma based on its anatomical location (3). Surgical resection is the preferred treatment for CCA in the early stage, whereas chemotherapy is the preferred treatment in the late stage; however, the overall survival rate with chemotherapy is low (4). Although great progress has been made in the diagnosis and treatment of CCA, its prognosis is still poor (5), and the 5-year survival rate of patients is less than 5%. Therefore, improving the early diagnosis of CCA could improve treatment efficacy and the patient survival rate (6). Primary sclerosis cholangitis is one of the most common risk factors for CCA; however, approximately 90% of patients have no identifiable risk factors (7). The clinical diagnosis of CCA includes clinical/biochemical features, imaging techniques, and serum nonspecific tumor biomarkers, which can assist in diagnosis; however, tumor biopsy is ultimately required to confirm the diagnosis (8). Currently, the clinical diagnosis of CCA is limited. Therefore, the application of biomarkers to stratify patients according to anatomical subtypes and genetic abnormalities is crucial for the development of targeted therapies (9). Gene chips can be used to rapidly detect differentially expressed genes (DEGs) in CCA, and this technology is reliable and has been used for more than a decade over a wide range of applications (10). The use of gene chip technology to explore potential genes closely related to the occurrence and development of CCA could reveal novel biomarkers for early diagnosis and potential targets for disease treatment, which would provide a basis for the further development of therapeutic drugs.

The Gene Expression Omnibus (GEO) is a database repository of high-throughput gene expression data, including gene chips, microarrays, and RNA-Seq data. It can be used in combination with bioinformatics tools to build biomarkers of interest (11). The Illumina BaseSpace Correlation Engine (BSCE) extends this research by integrating data collected by the GEO with the global genomics knowledge base. This interactive data analysis environment helps to validate results and test new hypotheses. Experimental molecular maps have been compared with the results of a large library of public/encrypted datasets using the BSCE to identify disease mechanisms, drug targets, and prognostic or predictive biomarkers; access web-based tool libraries; mine data; and create billions of new associations. Further, Gene Expression Profiling Interactive Analysis (GEPIA) is a web-based tool for the fast and customizable analyses of DNA and RNA sequencing expression data based on The Cancer Genome Atlas (TCGA) and Genotype-Tissue Expression (GTEx) databases.

## Materials and methods

### Microarray data

The NCBI-GEO is a free public database of microarray/gene-chip/RNA data. The sequences and gene expression profiles of GSE26566 (12), GSE31370 (13), and GSE77984 (14) based on CCA and normal bile duct tissues were obtained from GPL6104 (Illumina HumanRef-8 v2.0 Expression BeadChip) and GPL10558 (Illumina HumanGT-12 V4.0 Expression BeadChip). These include 104 CCA tissues and six normal bile duct tissues, five CCA tissues and five normal bile duct tissues, and four CCA tissues and four normal bile duct tissues, respectively.

### DEG data processing

Based on GEO2R online tools^1^, we used a |logFC| > 2 to identify DEGs between the bile duct carcinoma and normal bile duct tissue samples and adjusted the P-value to <0.05. Then, raw data in.txt format were filtered online using the Venn Diagrams database to detect DEGs among the three datasets. DEGs with a logFC < -2 were considered downregulated genes, whereas DEGs with a logFC > 2 were considered upregulated genes.

### Protein–protein interaction network and potential correlation analyses of DEGs

The arrangement and mapping of the PPI network provide an opportunity to further extract information on the evolutionary relationships among species through conservative pathways and protein complexes. The previously screened DEGs were inputted into the online STRING^2^ database to obtain the potential DEGs in the PPI network. The score was set to 0.8, and noninteracting protein molecules were hidden.

### Acquisition of clinical bile duct carcinoma DEGs

The Illumina BSCE^3^ is an RNA sequencing and microarray database that manages more than 23,000 scientific studies to provide data-driven gene, experimental, drug, and phenotypic expression information for research. “Cholangiocarcinoma”, “Homo sapiens”, and “RNA expression” were used as keywords to search curated studies of CCA to obtain GSEs. Ten biosets were selected for meta-analysis. In addition to the DEGs identified in the PPI network from the initial screening, nine other DEGs were selected based on their fold-change values across the 10 biosets and clinical significance in the literature.

### Correlation analysis and heat map generation with the GEO database

The 18 selected DEGs were inputted into the BSCE database for curative studies, one by one, using “cholangiocarcinoma”, “Homo sapiens”, and “RNA expression” as the keywords to build a template for correlation with CCA biosets using the Running Fisher test (15). The top-log (p-values) were inputted into Tree View version 1.6 to generate a heatmap to view the correlation.

### Verification of DEG expression

The GEPIA database was used to verify the differential expression of DEGs in clinical CCA between patients and normal controls with a large sample size.

### DEG correlation analysis

DEGs were inputted into GEPIA to obtain the correlation values between genes (18 DEGs). The results showed a significant correlation for the 28 combinations. Using intergenic R values, the correlation heatmap was drawn using the ballon plot/bubble plot tool of the Bioinformatics website. Finally, the correlation map of the 18 DEGs was established using the online STRING^4^ database.

### Immunohistochemistry

Tumor tissues and normal tissues adjacent to carcinoma were obtained from patients at the Affiliated Hospital of Zunyi Medical University (No. KLL-2022-619). Eleven patients were enrolled in this study. All procedures in this study were approved and implemented in accordance with the standards of the ethics committee of Zunyi Medical University Affiliated Hospital. The routine dewaxing method was used to detect the expression of FGA (1:100), OTC (1:100), CTH (1:100), and MMP11 (1:200) in all samples using immunohistochemistry. The experimental procedures were performed in strict accordance with the kit instructions. Finally, pathological changes were observed under an optical microscope (NI-U, Nikon, Japan), and images were captured using a digital camera (DS-RI2, Nikon). Image-pro Plus Version 6.0 software (Media Cybernetics, Inc., Rockville, MD, USA) was used to evaluate the area and density of the staining area, as well as the integrated optical density values of the immunohistochemical slices.

### Correlation analysis between CCA clinical biomarkers and hub genes

A PubMed search was performed with “cholangiocarcinoma” and “biomarkers” as keywords to search for a total of eight stage-specific and/or metastatic markers clinically relevant to CCA. The eight biomarkers were analyzed by correlation analysis with FGA, MMP11, OTC and CTH in the GEPIA database to obtain R values. Finally, using the R values between genes, we generated the correlation heatmap using the ballon plot/bubble plot tool of the Bioinformatics website.

### Statistical analysis

For the immunohistochemical analysis, the H-score method was used to conduct qualitative and quantitative analyses. All statistical calculations were performed using GraphPad Prism (version 6.01; GraphPad, San Diego, CA, USA). Statistical analysis of mean optical density data was performed using Student’s t-test to compare mean optical density differences between groups. P<0.05 was considered to indicate significance. All experiments were repeated more than three times.

## Results

### Identifications of DEGs in CCA

**Figure 1:**
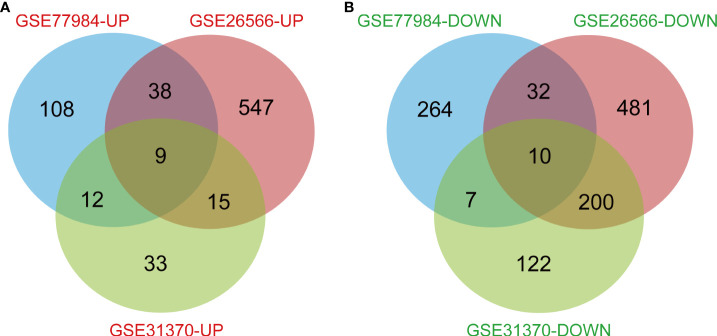
Genes that differed between normal subjects and CCA patients, including all intersecting genes in the three data sets. **(A)** 9 DEGs were upregulated (logFC>2). **(B)** 10 DEGs were downregulated (logFC<-2).

In this study, 113 cases of CCA and 15 normal bile ducts tissues were included. Using the GEO2R online tool, 1468, 520, and 580 DEGs were obtained from GSE26566, GSE31370, and GSE77984, respectively. The Venn Diagram online tool was then used to find the intersection of the three datasets. In total, 19 DEGs were screened based on the intersection of the three datasets, and they included 10 downregulated genes (logFC < -2) and nine upregulated genes (logFC > 2) (**Table 1** and **Figure 1**).

**Table 1:** 9 up-regulated and 10 down-regulated genes by Venn intersection.

DEGs	Genes name				
**Up-regulated**	C1orf106	C19orf33	EPCAM	LAMC2	KRT19
	PITX1	RAB25	SOX9	SPINT2	
**Down-regulated**	ADH1A	AOX1	C7	FGA	FGB
	FGG	HSD11B1	PRG4	SAA1	SLC7A2

### PPI and correlation analysis of potential DEGs

**Figure 2:**
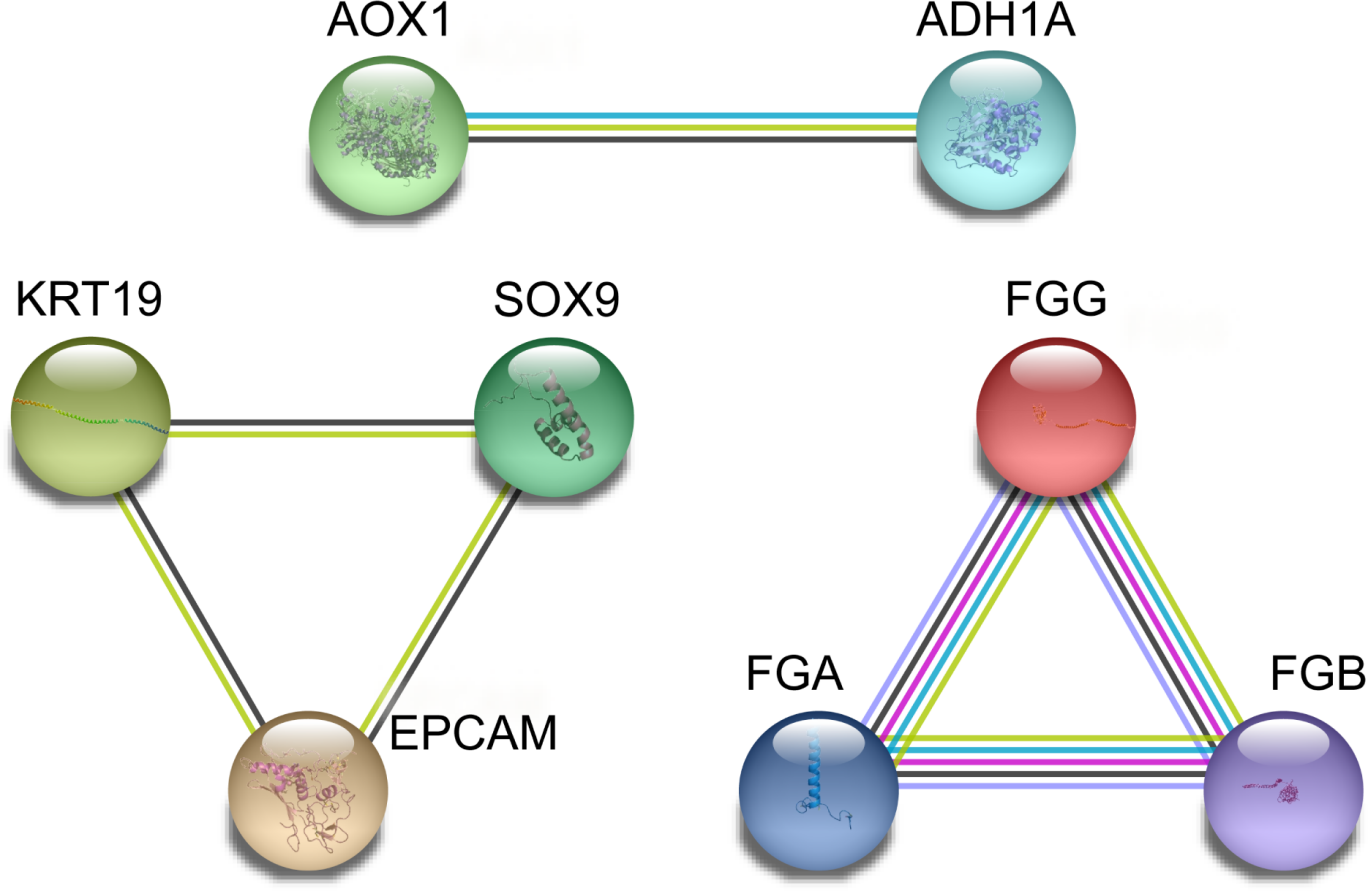
PPI network intersection of differential genes between the CCA patients and normal subjects was determined based on the STRING database.

Nineteen screened DEGs were imported into the STRING database to obtain the PPI network, and the score was set at 0.8 to remove unrelated genes. Eight DEGs were then selected (**Table 2** and **Figure 2**).

**Table 2:** The 8 DEGs obtained by STRING included 3 up-regulated and 5 down-regulated genes.

DEGs	Genes name				
**Up-regulated**	EPCAM	KRT19	SOX9		
**Down-regulated**	AOX1	ADH1A	FGA	FGB	FGG

### Selection of clinical bile duct carcinoma DEGs

**Figure 3:**
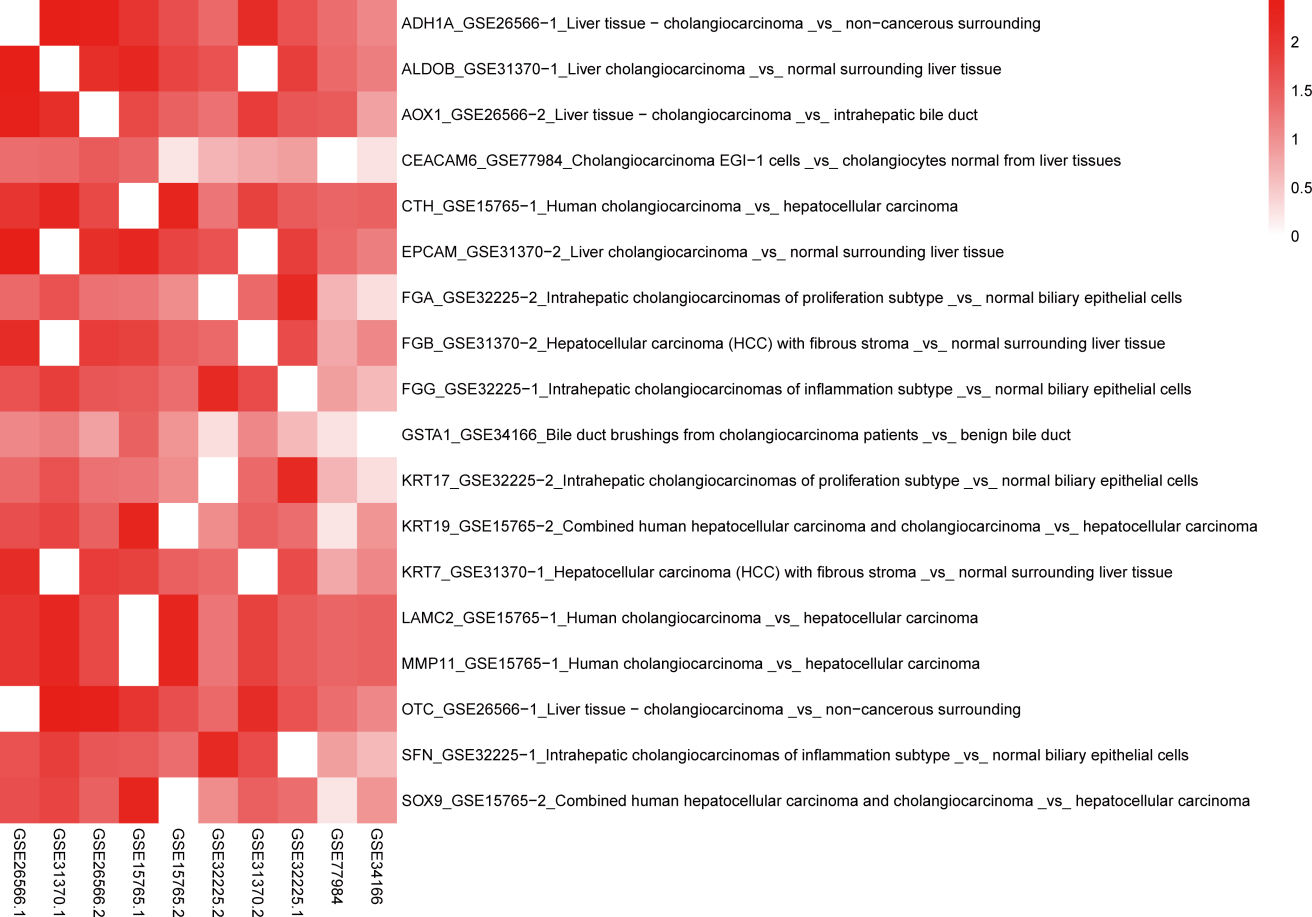
Heatmap of 18 DEGs and 10 biosets. Genes that correspond to the same biosets have a value of zero. A higher −log (p-values) corresponds to a higher positive correlation. The format is Gene_GSE bioset_Bioset name.

Using BSCE, 21 GSEs were obtained. GSEs from animals and cell culture studies were excluded, leaving six GSEs [GSE26566 (12), GSE31370 (13), GSE77984 (14), GSE32225 (16), GSE34166 (17), and GSE15765 (18)] containing 10 biosets (GSE26566-1, GSE26566-2, GSE31370-1, GSE31370-2, GSE77984, GSE32225-1, GSE32225-2, GSE15765-1, GSE15765-2, and GSE34166), as shown in the heatmap in **Figure 3**. These 10 biosets were subjected to a meta-analysis across all comparisons. In addition to the nine DEGs from the initial screen (including all eight in the PPI network), nine other DEGs were also selected based on their fold-change values and clinical significance from the literature, resulting in a total of 18 DEGs (**Table 3**). The 18 selected DEGs with clinical significance and the PMID numbers of related studies (up to five), are shown in **Table 4**.

**Table 3:** Selection of 9 up-regulated and 9 down-regulated DEGs.

DEGs	Genes name				
**Up-regulated**	CEACAM6	EPCAM	KRT7	KRT17	KRT19
	LAMC2	MMP11	SFN	SOX9	
**Down-regulated**	ADH1A	ALDOB	AOX1	CTH	GSTA1
	FGA	FGB	FGG	OTC	

**Table 4:** Selected DEGs with clinical relevance with PubMed ID.

DEGs	Genes name	PMID				
**Up-regulated**	CEACAM6	26974538	16868542	19055478	23806607	
	EPCAM	28267620	31450710	29497050	32984373	22907641
	KRT7	30588199	16258812	12588436	10320910	18622386
	KRT17	30953499	21333016	11003076		
	KRT19	27833076	31017316	30588199	30717258	31602368
	LAMC2	31345467	25773857	23775819	24124977	15105812
	MMP11	25987024	7632822			
	SFN	31751820	26729014	17391729	24030981	20976731
	SOX9	26341967	30420613	31383647	29464042	31128214
**Down-regulated**	ADH1A	31637215	27777109			
	ALDOB	31773848	32252311	32525595	27265876	33275593
	AOX1	31383940	31775891	25238417	18033820	
	CTH	28962123	31468690	33522955		
	FGA	22836734				
	FGB	31735647				
	FGG	30863175				
	GSTA1	24782056	31892975			
	OTC	30108309				

### Correlation analysis and heat map illustration of the GEO database

In total, 246 studies were extracted from the 18 DEGs to create a template for the correlation analysis with the 10 selected biosets using “Homo sapiens,” “RNA expression,” and “cholangiocarcinoma” as keywords in the BSCE database to generate −log(p-values). Higher −log(p-values) were indicative of a higher positive correlation. When the DEGs were correlated with the bioset derived from the −log(p-values), a value of 0 indicated a high correlation. For example, ADH1A was highly correlated with GSE26566-1 (0), GSE31370-1 (272), GSE26566-2 (220), GSE15765-1 (100), GSE15765-2 (48), GSE32225-1 (25), GSE31370-2 (137), GSE32225-1 (45), GSE77984 (22), and GSE34166 (12). Further, −log(p-values) >4 were considered significant (11). Thus, ADH1A was found to be a biomarker of CCA. The −log(p-values) for all 18 DEGs with 10 biosets are provided in **Supplementary Table 1** and can be easily visualized in the heatmap in **Figure 3**.

### Verification of DEG expression

**Figure 4:**
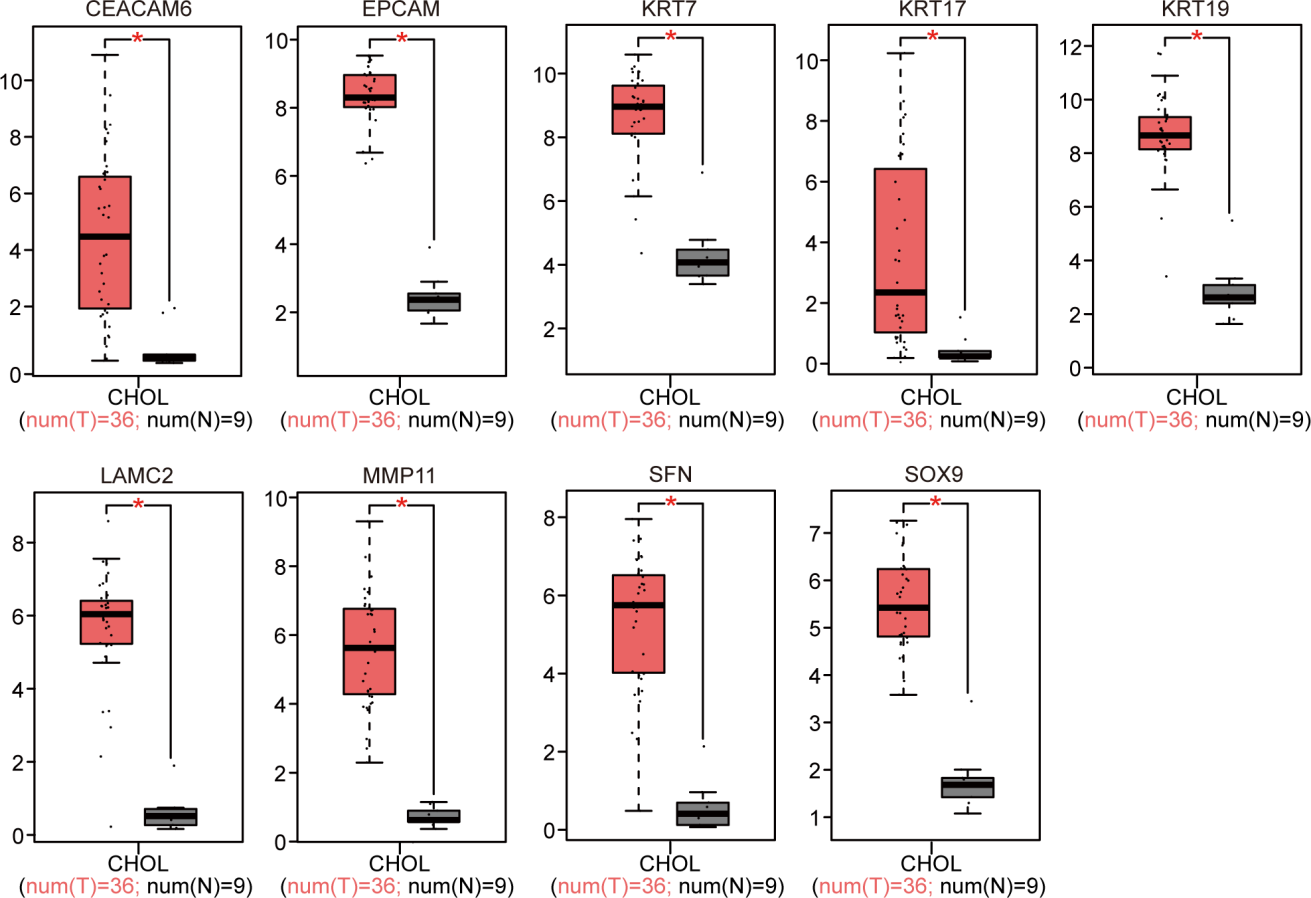
GEPIA website-based verification of the expression of nine upregulated genes associated with poor prognosis (*P < 0.05).

**Figure 5:**
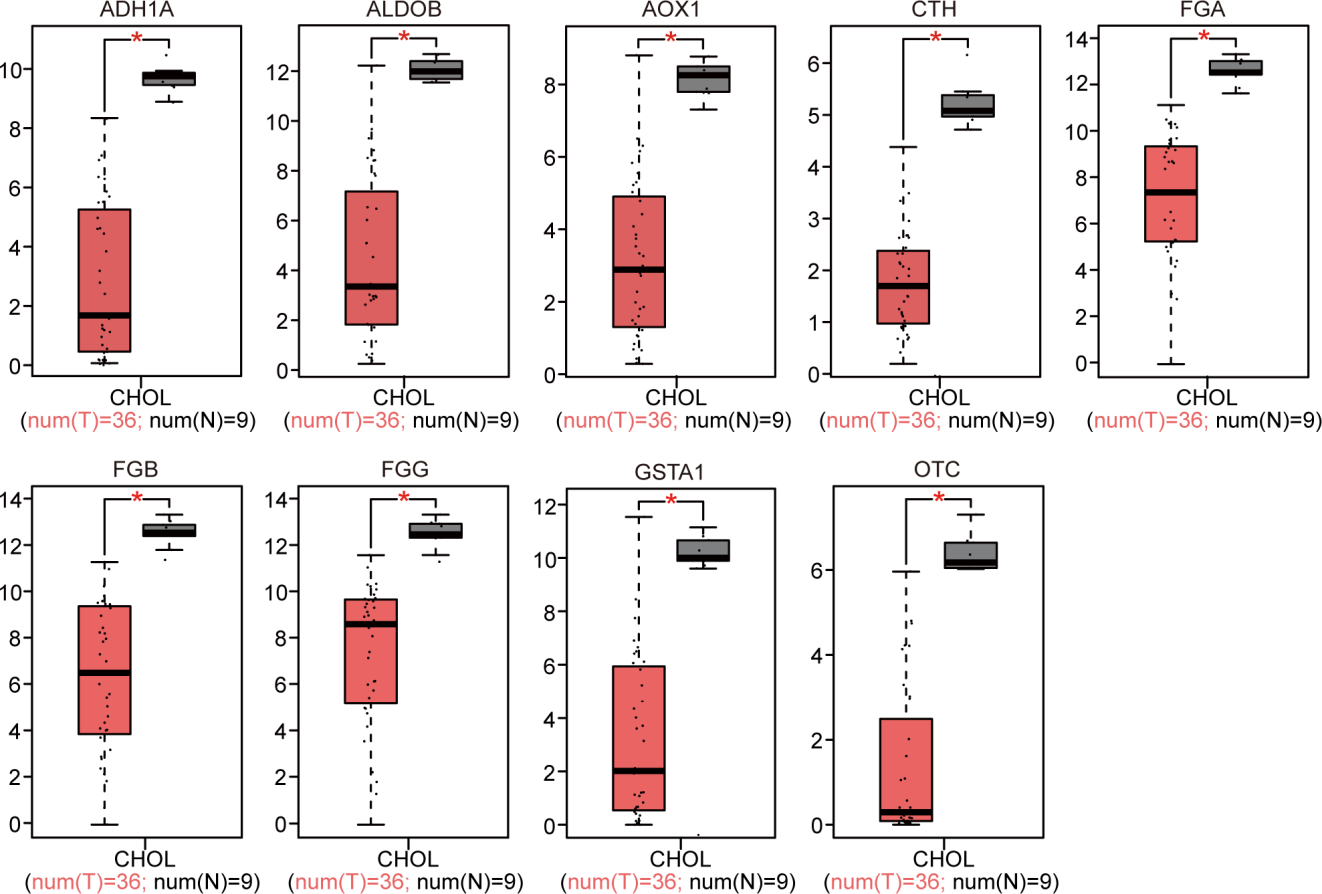
GEPIA website-based verification of the expression of nine downregulated genes associated with poor prognosis (*P < 0.05).

The 18 identified DEGs were inputted into GEPIA for the verification of differential expression between the CCA patients and normal controls based on a large sample size. The results showed that expression levels of nine genes were upregulated (P < 0.05, **Figure 4**), and those of nine genes were downregulated in the CCA patients (P < 0.05, **Figure 5**).

### Correlation analysis of DEGs

**Figure 6:**
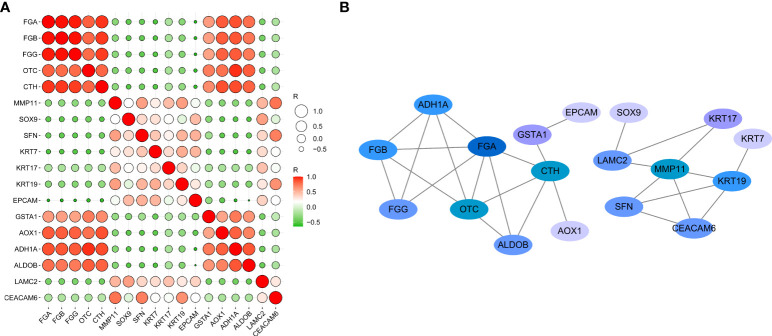
Network and heat map display correlation analysis of 18 gene expression in CCA. **(A)** Heatmap of correlation between genes. **(B)** The PPI network relationships of the gene combinations.

The correlations between DEGs (18 genes total, 153 combinations) were inputted into GEPIA for verification of the differential expression in CCA patients, based on a large sample size. The results showed that 28 combinations presented significant correlations (P < 0.05, **Supplementary Figure 1**). The R values between genes were inputted into the ballon plot/bubble plot tool of the Bioinformatics website to obtain the correlation heatmap. A more significant correlation between two genes was indicated by a darker corresponding color, with red indicating a positive correlation and green indicating a negative correlation (**Figure 6A**). The relationship built with STRING is shown in **Figure 6B**, where a connection between two genes indicates that the differential expression of the genes was significantly correlated in CCA patients, with a greater number of significantly related genes corresponding to a darker color.

### Identification of hub gene expression using immunohistochemistry

**Figure 7:**
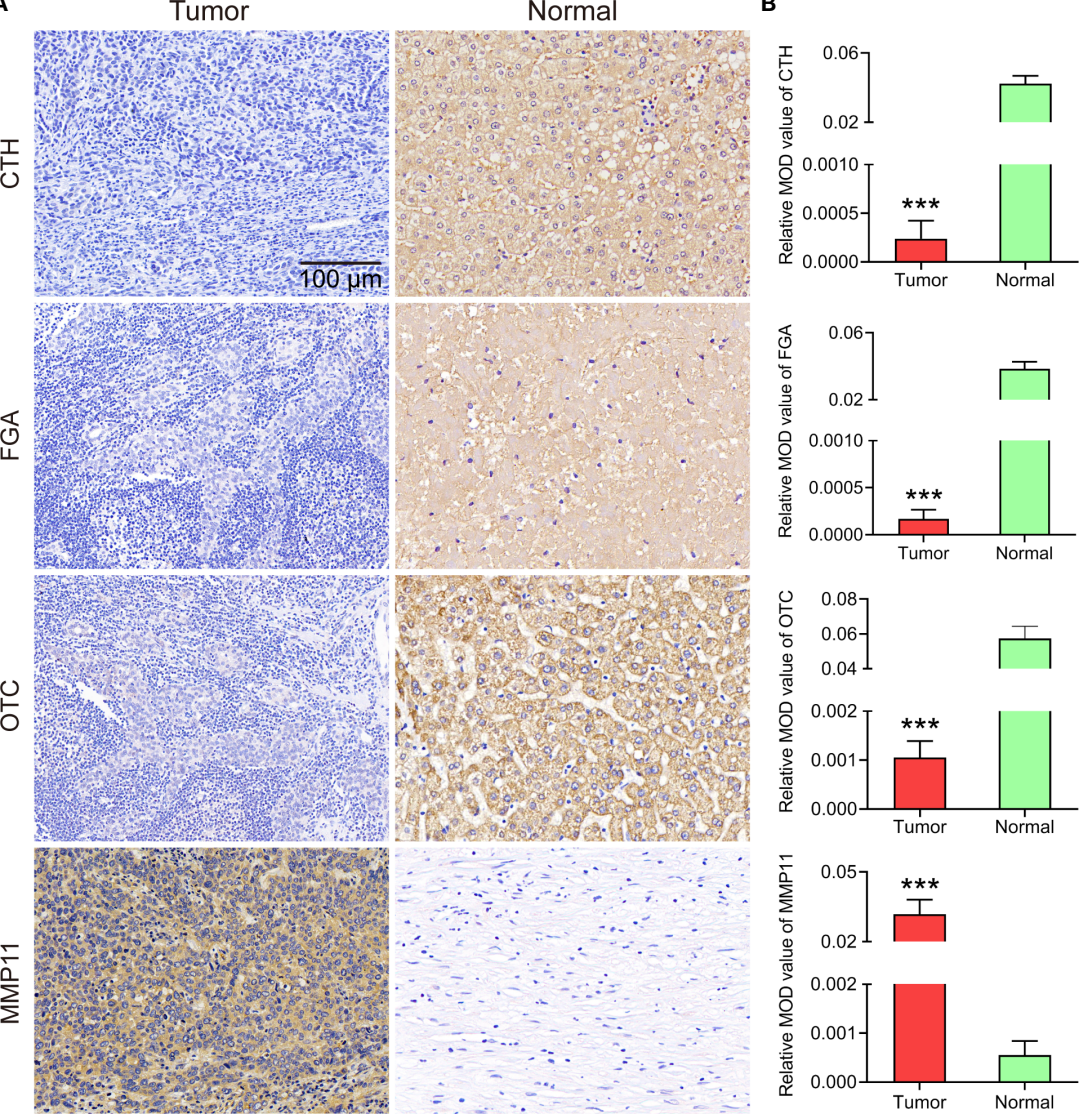
**(A)** Representative images of IHC staining from eleven primary CCA specimens and eleven paracancer normal tissue specimens (200x magnification). **(B)** Statistical analysis of the mean optical density (MOD) of CTH, FGA, OTC and MMP11 staining in CCA specimens and normal tissues. The bar chart shows the mean ±SEM of more than three independent experiments (***P< 0.001).

Immunohistochemical results were either positive or negative based on the H-score. The expression levels of *FGA*, *OTC*, *CTH*, and *MMP11* in each group are shown in **Figure 7**. Compared with that in normal tissues, CCA tissues had significantly lower *FGA*, *OTC*, and *CTH* expression but significantly higher *MMP11* expression.

### Correlations between hub genes and clinical biomarkers

**Figure 8:**
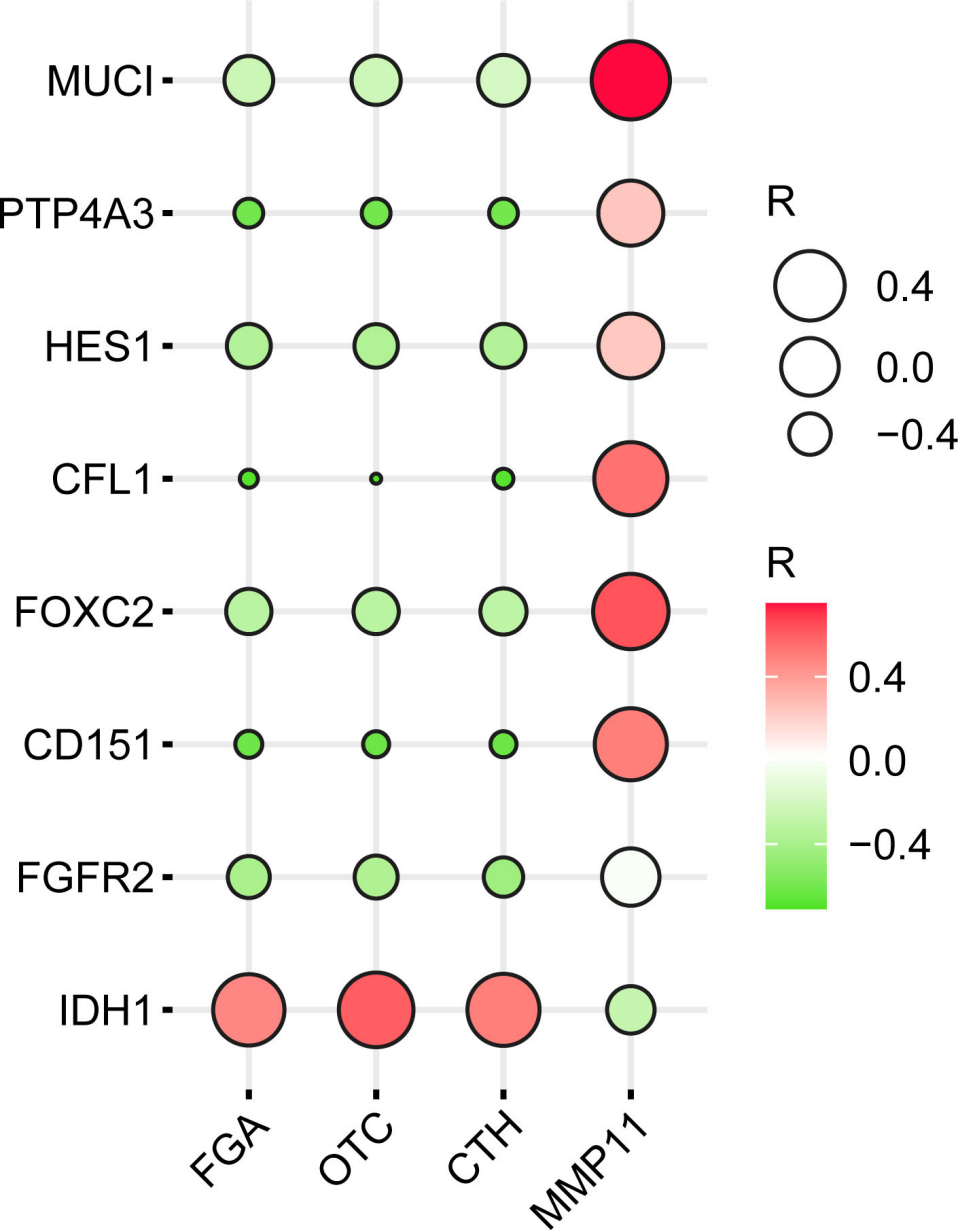
Heatmap of the correlation of (FGA, OTC, CTH, and MMP11) with clinical CCA stage-specific and/or metastatic markers.

The biomarkers specific for the clinical stage of CCA found in the PubMed were *MUCI*, *HES1*, and *CFL1*, among others, and the biomarkers related to metastasis were *PTP4A3*, *FOXC2*, *CD151*, *FGFR2*, and *IDH1*. R values obtained from the GEPIA database were derived from correlation values between four core genes and these eight genes. Inputted R values were plotted with the ballon plot/bubble plot tool for correlation heatmaps as shown in **Figure 8**. A more significant the correlation between two genes was indicated by a darker corresponding color, with red indicating a positive correlation and green indicating a negative correlation.

## Discussion

This study applied multiple bioinformatics tools (GEO2R and Venn) and available GEO databases to successfully screen and identify DEGs with protein interactions in PPI networks using STRING. We further utilized the Illumina BSCE to search curated studies on CCA and performed a literature search to identify an additional nine DEGs with clinical significance for CCA. Ultimately, 18 DEGs were highly correlated with the CCA gene expression database in the BSCE, and they were further verified with GEPIA for clinical significance. Four hub genes were screened and identified as potential diagnostic and therapeutic targets for CCA through comparisons between clinical CCA and normal tissues. This study is novel in that it clearly identifies CCA biomarkers and their clinical significance using available databases, and the findings might have a significant impact on early CCA diagnosis and treatment.

Most patients with CCA are diagnosed at an advanced stage and have poor overall survival. However, potential diagnostic and therapeutic targets of CCA remain unclear. In an attempt to identify more useful potential diagnostic and therapeutic targets and potential biomarkers for CCA, we initially utilized three NCBI-GEO datasets [GSE26566 (12), GSE31370 (13), and GSE77984 (14)] and collected the gene expression profiles of 113 CCA tissues and 15 normal bile duct tissues for bioinformatics analysis. Using the GEO2R and Venn Diagram tools, 18 DEGs were screened based on the criteria |logFC| > 2 and P < 0.05, and they included nine upregulated DEGs (logFC > 2) and nine downregulated DEGs (|logFC| < 2). These 18 DEGs were further imported into the STRING database to build the PPI network, and eight DEGs were identified that were significantly correlated with the expression profiles of CCA patients. Thus, GERO2R, Venn Diagram, and STRING are important bioinformatics tools used to identify CCA biomarkers and search molecular targets (19).

We further utilized the curated studies in the BSCE database (11) and selected additional GEO datasets (GSE32225 (16), GSE34166 (17), and GSE15765 (18)) containing 10 biosets with human CCA. Through the meta-analysis, the top and most consistent DEGs across these 10 biosets were selected and subjected to a literature search using “cholangiocarcinoma” as the keyword, and nine additional DEGs were identified. All selected DEGs had literature support (**Table 4**). GEPIA is a useful tool for determining the genomic features and clinical characteristics of CCA (20). We used GEPIA to verify the clinical significance of the 18 genes. The increased expression of *CEACAM6*, *EPCAM*, *LAMC2*, *MMP11*, *KRT7*, *KRT17*, *KRT19*, *SFN*, and *SOX9* (**Figure 4**) and the decreased expression of *ADH1A*, *ALDOB*, *AOX1*, *CTH*, *FGA*, *FGB*, *FGG*, *GSTA1*, and *OTC* (**Figure 5**) were significantly correlated and showed clinical significance for the diagnosis of CCA. These findings highlight the usefulness of the BSCE and GEPIA.

### Upregulated biomarkers in CCA

The human carcinoembryonic antigen-associated cell adhesion molecule 6 (*CEACAM6*) gene is highly expressed in CCA, and this molecule can be detected in the bile of CCA patients (21). *CEACAM6* is a potential prognostic indicator and probable drug resistance marker for intrahepatic CCA (22). Epithelial cell adhesion molecule (EPCAM) is a surface biomarker of tumor stem cells in bile duct carcinoma that is highly expressed in CCA (23, 24). EPCAM+ status, in combination with annexin V+ and ASGPR1+ fluorescence labeling, can be used as a noninvasive assessment of the presence and extent of CCA in patients with advanced liver diseases (24).

Cytokeratin19 (*KRT19*), *KRT17*, and *KRT7* are all members of the keratin gene family. *KRT19* is a specific and independent predictor of poor disease-free and overall survival in patients with CCA (25). *KRT19* plays an important role in the pathogenesis of CCA (26) and is a biomarker for the diagnosis of bile duct carcinoma (25–28). *KRT17* is mainly expressed in basal epithelial cells and many malignant tumors. *KRT17* immunostaining has been used to distinguish pancreatic head cancer from distal CCA (29, 30). *KRT7* expression is generally restricted to epithelia and neoplasms, and its rate of positivity in CCA is 93% (31). One case report indicated that ductal or glandular foci of iCCA present with strong and diffuse expression of *KRT7* (32).

High expression of laminin γ2 chain genes (*LAMC2*) in tumors is associated with CCA development *via* the EGFR signaling pathway. Silencing *LAMC2* inhibits EGFR activation and suppresses epithelial–mesenchymal transition in CCA (33). Matrix metalloproteinase-11 (*MMP11*) promotes the invasion and metastasis of CCA and is a biomarker for poor prognosis in this disease (34).

Stratified protein (SFN, 14-3-3σ) is highly expressed in detached CCA cells; silencing its expression results in increased CCA cell death and could be a potential target for CCA therapy (35). However, the overexpression of *SFN* was also reported to be associated with a better prognosis, lower early cancer recurrence rates, and distant metastasis following resection (35, 36). The transcription factor SOX9 is part of a superfamily of high-mobility domain transcription factors. The upregulation of *SOX9* expression reduces the expression of E-cadherin and increases the expression of vimentin and α-SMA, thereby promoting the invasion and metastasis of cancer cells (37–39) and decreasing the survival of patients with CCA by conferring chemoresistance (39).

### Downregulated biomarkers in CCA

Differential expression of the alcohol dehydrogenase 1A (*ADH1A*) gene has been observed in biliary stricture and liver cancer (40). *ADH1A* and aldehyde dehydrogenase 2 (*ALDH2*) are key regulators of alcohol metabolism, and their suppression aligns well with the gene expression profiles of hepatocellular carcinoma (HCC) and can predict HCC onset and progression. Based on the Liver Hepatocellular Carcinoma database in the Cancer Genome Atlas, higher expression levels of *ADH1A* and *ALDH2* are associated with good prognosis (41). Hepatic fructose-1, 6-diphosphate aldolase B (*ALDOB*) plays an important role in glycolysis. The loss of *ALDOB* activates Akt and promotes hepatocellular carcinogenesis by destabilizing the Aldob/Akt/PP2A protein complex (42). Risk factors for CCA include viral hepatitis, smoking, alcohol use, metabolic diseases, and genetic susceptibility (43). The suppressed expression of *ADH1A* and *ALDOB* could contribute to aberrant metabolism; thus, these genes represent a prognostic nutritional index for predicting survival and chemotherapy response in patients (44).

The relationship between decreased expression of aldehyde oxidase (*AOX1*) and cystathionine-γ-lyase (*CTH*) and the prognosis of patients with CCA has not been previously reported. *AOX1* catalyzes the oxidation of a variety of endogenous and exogenous aldehydes and N-heterocyclic aromatic compounds, and the epigenetic loss of *AOX1* leads to metabolic deregulation and promotes cancer progression (45). *AOX1* is a DNA methylation marker for prostate cancer, and hypermethylation leads to *AOX1* inhibition (46). Similarly, DNA hypermethylation of the *CTH* promoter caused by the *FOXC1*-mediated upregulation of DNA methylase 3B results in low *CTH* expression in HCC cells, and HCC patients with positive *FOXC1* expression and negative *CTH* expression exhibit the worst prognosis (47).

Fibrinogens consist of three pairs of polypeptide chains that are not identical, and different genes encode fibrinogen α (*FGA*), fibrinogen β (*FGB*), and fibrinogen γ (*FGG*) (48). *FGA* is enhanced by mutations in fibrinogen enhancer 2. Genome sequence variations may affect the production of liver fibrinogen and enhance expression in liver cancer cells (49). *FGB* and *CRP* are inflammatory markers in iCCA samples, and reduced inflammatory marker scores in the periductal-infiltrative type, mass-forming type without the cholangiolocellular differentiation trait, and non-small-duct type iCCAs are associated with poor prognosis (50). *FGG* is expressed in primary liver cancer tissues, and clinicopathological analyses have shown that the upregulation of intracellular *FGG* expression is correlated with an increase in tumor vascular infiltration. HCC patients with upregulated *FGG* expression have a higher recurrence rate and a correspondingly shorter overall survival time (51). Ten serum-derived exosomal proteins were screened as potential biomarkers for HCC; the expression of six of these was downregulated (including *FGA*, *FGB*, and *FGG*) at the protein level, which was verified at the gene expression level through GEPIA (52), which is consistent with the current findings.

Glutathione S-transferases (GSTs) play an important role in detoxification, metabolism, and carcinogenesis. Lower *GSTA1* expression is associated with poor prognosis in patients with HCC (53), which is consistent with the reduced *GSTA1* expression in patients with CCA in the present study. GSE34166 (17) was used to build biomarkers for CCA, with *GSTA1* representing the most critical hub gene and a *GSTA1*-based interaction network consisting of 25 genes, including *GSTA3* (54).

Ornithine carbamoyltransferase (OTC) is a metabolizing enzyme responsible for converting nitrogenous waste into urea and allowing for its excretion. When the urea cycle is impaired, ammonia accumulates in the blood, resulting in toxicity and tumorigenesis (55). Hepatocellular neoplasms can develop with genetic metabolic disorders, such as OTC deficiency (56). In the present study, reduced *OTC* expression was found to be associated with CCA.

CCA is a cancer of the intrahepatic or extrahepatic bile duct that is characterized by a late diagnosis, high mortality, and early invasion and metastasis. The occurrence and development of CCA are associated with biliary tract inflammation, cholestasis, and liver inflammation (57). The 18 genes identified in this study could serve as potential prognostic biomarkers for the diagnosis and treatment of CCA. The PPI network analysis indicated that the expression of these 18 protein-encoding genes was significantly correlated with their integrated roles in CCA. The GEPIA database was used to screen these 18 genes, resulting in the identification of four genes with the highest correlation, and clinical samples were used to verify their expression. The data obtained in this study might provide useful information and directions for further research on CCA. However, further research is warranted to improve the prognosis of patients with these rare tumors. However, in this study, these four hub genes were only screened using a database, and were validated using the tissues of 11 clinical cholangiocarcinoma patients. Expression in CCA also needs to be validated by performing both *in vivo* and *in vitro* experiments and should be further investigated with a large patient sample size. Currently existing tumor biomarkers have poor specificity and low sensitivity, are associated with false positives and negatives, and lack of predictivity, consistency, and standardization. In addition, different research groups use different databases to screen different tumors, or use different methods to study different tumors, which has resulted in massive amounts of data, with no accurate conclusions, and the possibility of accidental error, among other issues.

In summary, the bioinformatics analysis performed in this study utilized the GEO database and the GEO2R, Venn diagram, STRING, BSCE, and GEPIA tools to identify and verify 18 biomarkers of CCA. The 18 biomarkers were analyzed by GEPIA; four hub genes with the highest correlations (*FGA*, *OTC*, *CTH*, and *MMP11*) were screened, and their expression was verified by immunohistochemistry. The results showed that the expression of *FGA*, *OTC*, and *CTH* in the tumor tissues of patients with CCA is significantly lower than that in normal tissues, but that the expression of *MMP11* is significantly higher than that in normal tissues. The experimental results were consistent with those of the bioinformatics and meta-analyses. These four hub genes might play key roles in the occurrence and development of CCA and could serve as potential biomarkers of this disease, which could improve its diagnosis and treatment, as well as patient prognosis. The screening of tumor markers preliminarily confirmed that these four genes were related to the occurrence of CCA; however, quantitative studies have not been performed, and the results presented here should be verified using a large sample size based on their detection in each CCA stage.

## Data availability statement

Publicly available datasets were analyzed in this study. This data can be found here: www.ncbi.nlm.nih.gov/geo/. The NCBI-GEO is a free public database of microarray/gene-chip/RNA data. The sequence and gene expression profiles of GSE26566, GSE31370, and GSE77984 in CCA and normal bile duct tissues were obtained from GPL6104 (Illumina HumanRef-8 v2.0 Expression BeadChip) and GPL10558 (Illumina HumanGT-12 V4.0 Expression BeadChip). These included 104 CCA tissues and 6 normal bile duct tissues, 5 CCA tissues and 5 normal bile duct tissues, and 4 CCA tissues and 4 normal bile duct tissues, respectively.

## Ethics statement

This study was reviewed and approved by All procedures in this study were approved and implemented in accordance with the standards of the ethics committee of Zunyi Medical University Affiliated Hospital. Written informed consent for participation was not required for this study in accordance with the national legislation and the institutional requirements.

## Author contributions

(I) Conception and design of the study: PL and TZ; (II) Analysis and interpretation of data: HS, PL, XB, JL; (III) Drafting the article or revising it critically for important intellectual content: PL, XB, JL and TZ; (IV) Final approval of the version to be submitted: all authors; (V) Reviewer response: HS. All authors contributed to the article and approved the submitted version.

## Funding

This work was supported by the Program for Excellent Young Talents of Zunyi Medical University [Grant No.18zy-006] and the Basic Research Program of Guizhou Province, Department of Science and Technology (Natural Science) [Grant No. qiankehejichu-ZK [2022]yiban599].

## Acknowledgments

We would like to thank Editage (accounts.editage.cn/) for English Language editing.

## Conflict of interest

The authors declare that the research was conducted in the absence of any commercial or financial relationships that could be construed as a potential conflict of interest.

## Publisher’s note

All claims expressed in this article are solely those of the authors and do not necessarily represent those of their affiliated organizations, or those of the publisher, the editors and the reviewers. Any product that may be evaluated in this article, or claim that may be made by its manufacturer, is not guaranteed or endorsed by the publisher.

## Footnotes

1
www.ncbi.nlm.nih.gov/geo/geo2r/

2
string-db.org/

3
login.illumina.com/platform-services-manager/?rURL=//epa.ce.

4
string-db.org/

## Supplementary material

Supplementary Figure 1 Correlation analysis of DEGs expression in patients with CCA.

Supplementary Figure 2 Immunohistochemical results of CTH, FGA, OTC and MMP11 of 10 CCA samples other than **Figure 7**.

## Abbreviations

ADH1A, alcohol dehydrogenase 1A; ALDH2, aldehyde dehydrogenase 2; ALDOB, hepatic fructose-1, 6-diphosphate aldolase B; AOX1, aldehyde oxidase; BSCE, Illumina Base Space Correlation Engine; CCA, cholangiocarcinoma; CTH, cystathionin-γ-lyase; DEG, differentially expressed gene; EPCAM, epithelial cell adhesion molecule; GEO, gene expression omnibus; GEPIA, gene expression profiling interactive analysis; GST, glutathione S-transferase; HCC, hepatocellular carcinoma; iCCA, intrahepatic cholangiocarcinoma; KRT, cytokeratin; LAMC2, laminin γ2 chain; MMP11, matrix metalloproteinase-11; OTC, ornithine carbamyl transferase; PPI, protein-protein interaction.

## References

[1] Blechacz B. Cholangiocarcinoma: Current knowledge and new developments. Gut Liver (2017) 11(1):13–26. doi: 10.5009/gnl15568 27928095 PMC5221857

[2] Khan AS Dageforde LA. Cholangiocarcinoma. Surg Clin North Am (2019) 99(2):315–35. doi: 10.1016/j.suc.2018.12.004 30846037

[3] Rizvi S Khan SA Hallemeier CL Kelley RK Gores GJ. Cholangiocarcinoma - evolving concepts and therapeutic strategies. Nat Rev Clin Oncol (2018) 15(2):95–111. doi: 10.1038/nrclinonc.2017.157 28994423 PMC5819599

[4] Brindley PJ Bachini M Ilyas SI. Cholangiocarcinoma. Nat Rev Dis Primers (2021) 7(1):65. doi: 10.1038/s41572-021-00300-2. 34504109 PMC9246479

[5] Chong DQ Zhu AX. The landscape of targeted therapies for cholangiocarcinoma: current status and emerging targets. Oncotarget (2016) 7(29):46750–67. doi: 10.18632/oncotarget.8775 PMC5216834 27102149

[6] Grimsrud MM Folseraas T. Pathogenesis, diagnosis and treatment of premalignant and malignant stages of cholangiocarcinoma in primary sclerosing cholangitis. Liver Int (2019) 39(12):2230–7. doi: 10.1111/liv.14180 31216595

[7] Clements O Eliahoo J Kim JU Taylor-Robinson SD Khan SA. Risk factors for intrahepatic and extrahepatic cholangiocarcinoma: A systematic review and meta-analysis. J Hepatol (2020) 72(1):95–103. doi: 10.1016/j.jhep.2019.09.007 31536748

[8] Nakano M Ariizumi SI Yamamoto M. Intrahepatic cholangiocarcinoma. Semin Diagn Pathol (2017) 34(2):160–6. doi: 10.1053/j.semdp.2016.12.012 28094162

[9] Qian F Guo J Jiang Z Shen B. Translational bioinformatics for cholangiocarcinoma: Opportunities and challenges. Int J Biol Sci (2018) 14(8):920–9. doi: 10.7150/ijbs.24622 PMC6036745 29989102

[10] Jia W Zhang Y Guo Q Sun Q Guo Q Ji Z. [Application of gene chip technology for acupuncture research over the past 15 years]. Zhongguo Zhen jiu = Chin Acupuncture Moxibustion (2017) 37(12):1358–62. doi: 10.13703/j.0255-2930.2017.12.029 29355005

[11] Corton JC Kleinstreuer NC Judson RS. Identification of potential endocrine disrupting chemicals using gene expression biomarkers. Toxicol Appl Pharmacol (2019) 380:114683. doi: 10.1016/j.taap.2019.114683 31325560

[12] Andersen JB Spee B Blechacz BR Avital I Komuta M Barbour A. Genomic and genetic characterization of cholangiocarcinoma identifies therapeutic targets for tyrosine kinase inhibitors. Gastroenterology (2012) 142(4):1021–1031.e15. doi: 10.1053/j.gastro.2011.12.005 22178589 PMC3413201

[13] Seok JY Na DC Woo HG Roncalli M Kwon SM Yoo JE. A fibrous stromal component in hepatocellular carcinoma reveals a cholangiocarcinoma-like gene expression trait and epithelial-mesenchymal transition. Hepatology (2012) 55(6):1776–86. doi: 10.1002/hep.25570 22234953

[14] Merino-Azpitarte M Lozano E Perugorria MJ Esparza-Baquer A Erice O Santos-Laso Á. SOX17 regulates cholangiocyte differentiation and acts as a tumor suppressor in cholangiocarcinoma. J Hepatol (2017) 67(1):72–83. doi: 10.1016/j.jhep.2017.02.017 28237397 PMC5502751

[15] Kupershmidt I Su QJ Grewal A Sundaresh S Halperin I Flynn J. Ontology-based meta-analysis of global collections of high-throughput public data. PloS One (2010) 5(9):e13066. doi: 10.1371/journal.pone.0013066 20927376 PMC2947508

[16] Sia D Hoshida Y Villanueva A Roayaie S Ferrer J Tabak B. Integrative molecular analysis of intrahepatic cholangiocarcinoma reveals 2 classes that have different outcomes. Gastroenterology (2013) 144(4):829–40. doi: 10.1053/j.gastro.2013.01.001 PMC3624083 23295441

[17] Chapman MH Tidswell R Dooley JS Sandanayake NS Cerec V Deheragoda M. Whole genome RNA expression profiling of endoscopic biliary brushings provides data suitable for biomarker discovery in cholangiocarcinoma. J Hepatol (2012) 56(4):877–85. doi: 10.1016/j.jhep.2011.10.022 PMC3307884 22173169

[18] Woo HG Lee JH Yoon JH Kim CY Lee HS Jang JJ. Identification of a cholangiocarcinoma-like gene expression trait in hepatocellular carcinoma. Cancer Res (2010) 70(8):3034–41. doi: 10.1158/0008-5472.CAN-09-2823 PMC3498758 20395200

[19] Yang P He H Xu S Liu P Bai X. Potential molecular target prediction and docking verification of hua-Feng-Dan in stroke based on network pharmacology. Evid Based Complement Alternat Med (2020) 2020:8872593. doi: 10.1155/2020/8872593 33193801 PMC7641700

[20] Feng H Tong H Yan J He M Chen W Wang J. Genomic features and clinical characteristics of adolescents and young adults with cholangiocarcinoma. Front Oncol (2020) 9:1439. doi: 10.3389/fonc.2019.01439 32010606 PMC6971196

[21] Rose JB Correa-Gallego C Li Y Nelson J Alseidi A Helton WS. The role of biliary carcinoembryonic antigen-related cellular adhesion molecule 6 (CEACAM6) as a biomarker in cholangiocarcinoma. PloS One (2016) 11(3):e0150195. doi: 10.1371/journal.pone.0150195 26974538 PMC4790932

[22] Ieta K Tanaka F Utsunomiya T Kuwano H Mori M. CEACAM6 gene expression in intrahepatic cholangiocarcinoma. Br J Cancer (2006) 95(4):532–40. doi: 10.1038/sj.bjc.6603276 PMC2360665 16868542

[23] Wu HJ Chu PY. Role of cancer stem cells in cholangiocarcinoma and therapeutic implications. Int J Mol Sci (2019) 20(17):4154. doi: 10.3390/ijms20174154 PMC6747544 31450710

[24] Julich-Haertel H Urban SK Krawczyk M Willms A Jankowski K Patkowski W. Cancer-associated circulating large extracellular vesicles in cholangiocarcinoma and hepatocellular carcinoma. J Hepatol (2017) 67(2):282–92. doi: 10.1016/j.jhep.2017.02.024 28267620

[25] Akita M Ajiki T Fukumoto T Itoh T Zen Y. Keratin 19-expressing hepatocellular carcinoma and small-duct type intrahepatic cholangiocarcinoma show a similar postoperative clinical course but have distinct genetic features. Histopathology (2019) 75(3):385–93. doi: 10.1111/his.13884 31017316

[26] Wang P Lv L. miR-26a induced the suppression of tumor growth of cholangiocarcinoma *via* KRT19 approach. Oncotarget (2016) 7(49):81367–76. doi: 10.18632/oncotarget.13229 PMC5348398 27833076

[27] Yokomichi N Nishida N Umeda Y Taniguchi F Yasui K Toshima T. Heterogeneity of epigenetic and epithelial mesenchymal transition marks in hepatocellular carcinoma with keratin 19 proficiency. Liver Cancer (2019) 8(4):239–54. doi: 10.1159/000490806 PMC6738240 31602368

[28] Van Haele M Moya IM Karaman R Rens G Snoeck J Govaere O. YAP and TAZ heterogeneity in primary liver cancer: An analysis of its prognostic and diagnostic role. Int J Mol Sci (2019) 20(3):638. doi: 10.3390/ijms20030638 PMC6386931 30717258

[29] Seol MA Chu IS Lee MJ Yu GR Cui XD Cho BH. Genome-wide expression patterns associated with oncogenesis and sarcomatous transdifferentation of cholangiocarcinoma. BMC Cancer (2011) 11:78. doi: 10.1186/1471-2407-11-78 21333016 PMC3053267

[30] Takenami T Maeda S Karasawa H Suzuki T Furukawa T Morikawa T. Novel biomarkers distinguishing pancreatic head cancer from distal cholangiocarcinoma based on proteomic analysis. BMC Cancer (2019) 19(1):318. doi: 10.1186/s12885-019-5548-x 30953499 PMC6451218

[31] Chu P Wu E Weiss LM. Cytokeratin 7 and cytokeratin 20 expression in epithelial neoplasms: a survey of 435 cases. Mod Pathol (2000) 13(9):962–72. doi: 10.1038/modpathol.3880175 11007036

[32] Tsuneyama K Kaizaki Y Doden K Kidani E Harada K Sasaki M. Combined hepatocellular and cholangiocarcinoma with marked squamous cell carcinoma components arising in non-cirrhotic liver. Pathol Int (2003) 53(2):90–7. doi: 10.1046/j.1440-1827.2003.01443.x 12588436

[33] Pei YF Liu J Cheng J Wu WD Liu XQ. Silencing of LAMC2 reverses epithelial-mesenchymal transition and inhibits angiogenesis in cholangiocarcinoma *via* inactivation of the epidermal growth factor receptor signaling pathway. Am J Pathol (2019) 189(8):1637–53. doi: 10.1016/j.ajpath.2019.03.012 31345467

[34] Tongtawee T Kaewpitoon SJ Loyd R Chanvitan S Leelawat K Praditpol N. High expression of matrix metalloproteinase-11 indicates poor prognosis in human cholangiocarcinoma. Asian Pac J Cancer Prev (2015) 16(9):3697–701. doi: 10.7314/apjcp.2015.16.9.3697 25987024

[35] Khongmanee A Lirdprapamongkol K Tit-oon P Chokchaichamnankit D Svasti J Srisomsap C. Proteomic analysis reveals important role of 14-3-3σ in anoikis resistance of cholangiocarcinoma cells. Proteomics (2013) 13(21):3157–66. doi: 10.1002/pmic.201300219 24030981

[36] Sirivatanauksorn V Dumronggittigule W Dulnee B Srisawat C Sirivatanauksorn Y Pongpaibul A. Role of stratifin (14-3-3 sigma) in adenocarcinoma of gallbladder: A novel prognostic biomarker. Surg Oncol (2020) 32:57–62. doi: 10.1016/j.suronc.2019.10.022 31751820

[37] He J Gerstenlauer M Chan LK Leithäuser F Yeh MM Wirth T. Block of NF-kB signaling accelerates MYC-driven hepatocellular carcinogenesis and modifies the tumor phenotype towards combined hepatocellular cholangiocarcinoma. Cancer Lett (2019) 458:113–22. doi: 10.1016/j.canlet.2019.05.023 31128214

[38] Matsushima H Kuroki T Kitasato A Adachi T Tanaka T Hirabaru M. Sox9 expression in carcinogenesis and its clinical significance in intrahepatic cholangiocarcinoma. Dig Liver Dis (2015) 47(12):1067–75. doi: 10.1016/j.dld.2015.08.003 26341967

[39] Yuan X Li J Coulouarn C Lin T Sulpice L Bergeat D. SOX9 expression decreases survival of patients with intrahepatic cholangiocarcinoma by conferring chemoresistance. Br J Cancer (2018) 119(11):1358–66. doi: 10.1038/s41416-018-0338-9 PMC6265288 30420613

[40] Gupta MK Behara SK Vadde R. In silico analysis of differential gene expressions in biliary stricture and hepatic carcinoma. Gene (2017) 597:49–58. doi: 10.1016/j.gene.2016.10.032 27777109

[41] Zahid KR Yao S Khan ARR Raza U Gou D. mTOR/HDAC1 crosstalk mediated suppression of ADH1A and ALDH2 links alcohol metabolism to hepatocellular carcinoma onset and progression in silico. Front Oncol (2019) 9:1000. doi: 10.3389/fonc.2019.01000 31637215 PMC6787164

[42] He X Li M Yu H Liu G Wang N Yin C. Loss of hepatic aldolase b activates akt and promotes hepatocellular carcinogenesis by destabilizing the Aldob/Akt/PP2A protein complex. PloS Biol (2020) 18(12):e3000803. doi: 10.1371/journal.pbio.3000803 33275593 PMC7744066

[43] Massarweh NN El-Serag HB. Epidemiology of hepatocellular carcinoma and intrahepatic cholangiocarcinoma. Cancer Control. (2017) 24(3):1073274817729245. doi: 10.1177/1073274817729245 28975830 PMC5937247

[44] Salati M Filippi R Vivaldi C Caputo F Leone F Salani F. The prognostic nutritional index predicts survival and response to first-line chemotherapy in advanced biliary cancer. Liver Int (2020) 40(3):704–11. doi: 10.1111/liv.14314 31773848

[45] Vantaku V Putluri V Bader DA Maity S Ma J Arnold JM. Epigenetic loss of AOX1 expression *via* EZH2 leads to metabolic deregulations and promotes bladder cancer progression. Oncogene (2020) 39(40):6265–85. doi: 10.1038/s41388-019-0902-7 PMC8058741 31383940

[46] Strand SH Orntoft TF Sorensen KD. Prognostic DNA methylation markers for prostate cancer. Int J Mol Sci (2014) 15(9):16544–76. doi: 10.3390/ijms150916544 PMC4200823 25238417

[47] Lin Z Huang W He Q Li D Wang Z Feng Y. FOXC1 promotes HCC proliferation and metastasis by upregulating DNMT3B to induce DNA hypermethylation of CTH promoter. J Exp Clin Cancer Res (2021) 40(1):50. doi: 10.1186/s13046-021-01829-6 33522955 PMC7852227

[48] Bahadori B Uitz E Dehchamani D Pilger E Renner W. The fibrinogen gamma 10034C>T polymorphism is not associated with peripheral arterial disease. Thromb Res (2010) 126(4):350–2. doi: 10.1016/j.thromres.2010.07.007 20709368

[49] Fish RJ Neerman-Arbez M. A novel regulatory element between the human FGA and FGG genes. Thromb Haemost. (2012) 108(3):427–34. doi: 10.1160/TH12-04-0274 22836734

[50] Chung T Rhee H Nahm JH Jeon Y Yoo JE Kim YJ. Clinicopathological characteristics of intrahepatic cholangiocarcinoma according to gross morphologic type: cholangiolocellular differentiation traits and inflammation- and proliferation-phenotypes. HPB (Oxford). (2020) 22(6):864–73. doi: 10.1016/j.hpb.2019.10.009 31735647

[51] Zhang X Wang F Huang Y Ke K Zhao B Chen L. FGG promotes migration and invasion in hepatocellular carcinoma cells through activating epithelial to mesenchymal transition. Cancer Manag Res (2019) 11:1653–65. doi: 10.2147/CMAR.S188248 PMC6389006 30863175

[52] Zhao L Shi J Chang L Wang Y Liu S Li Y. Serum-derived exosomal proteins as potential candidate biomarkers for hepatocellular carcinoma. ACS Omega. (2021) 6(1):827–35. doi: 10.1021/acsomega.0c05408 PMC7808137 33458533

[53] Liu X Sui X Zhang C Wei K Bao Y Xiong J. Glutathione s-transferase A1 suppresses tumor progression and indicates better prognosis of human primary hepatocellular carcinoma. J Cancer (2020) 11(1):83–91. doi: 10.7150/jca.36495 31892975 PMC6930411

[54] Qi DC Wu B Tao SL Zhou J Qian HX Wang D. Analysis of differentially expressed genes in malignant biliary strictures. Genet Mol Res (2014) 13(2):2674–82. doi: 10.4238/2014.April.8.10 24782056

[55] Alexandrou C Al-Aqbi SS Higgins JA Boyle W Karmokar A Andreadi C. Sensitivity of colorectal cancer to arginine deprivation therapy is shaped by differential expression of urea cycle enzymes. Sci Rep (2018) 8(1):12096. doi: 10.1038/s41598-018-30591-7 30108309 PMC6092409

[56] Cheng L Jain D Kakar S Torbenson MS Wu TT Yeh MM. Hepatocellular neoplasms arising in genetic metabolic disorders: steatosis is common in both the tumor and background liver. Hum Pathol (2021) 108:93–9. doi: 10.1016/j.humpath.2020.11.012 33245984

[57] Mar WA Shon AM Lu Y Yu JH Berggruen SM Guzman G. Imaging spectrum of cholangiocarcinoma: role in diagnosis, staging, and posttreatment evaluation. Abdom Radiol (NY) (2016) 41(3):553–67. doi: 10.1007/s00261-015-0583-9 26847022

