# Supplementary figures and images for: Screening potential biomarkers of cholangiocarcinoma based on gene chip meta-analysis and small-sample experimental research
Source: Front Oncol. 2022 Oct 10;12:1001400. doi: 10.3389/fonc.2022.1001400 (PMC9590411; doi:10.3389/fonc.2022.1001400)

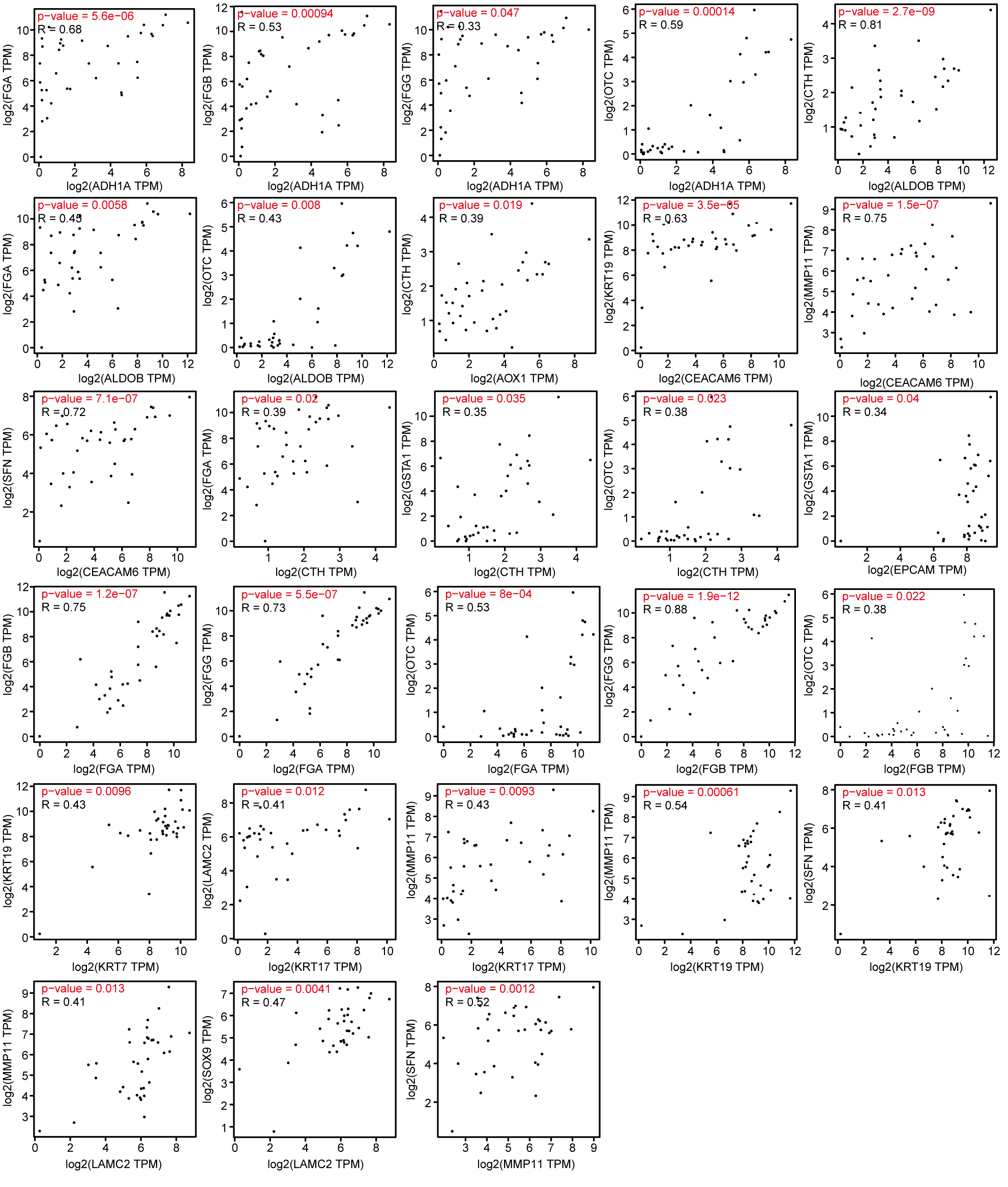

Supplement: Supplementary Figure 1 — Correlation analysis of DEGs expression in patients with CCA. [file Image_1.tif]

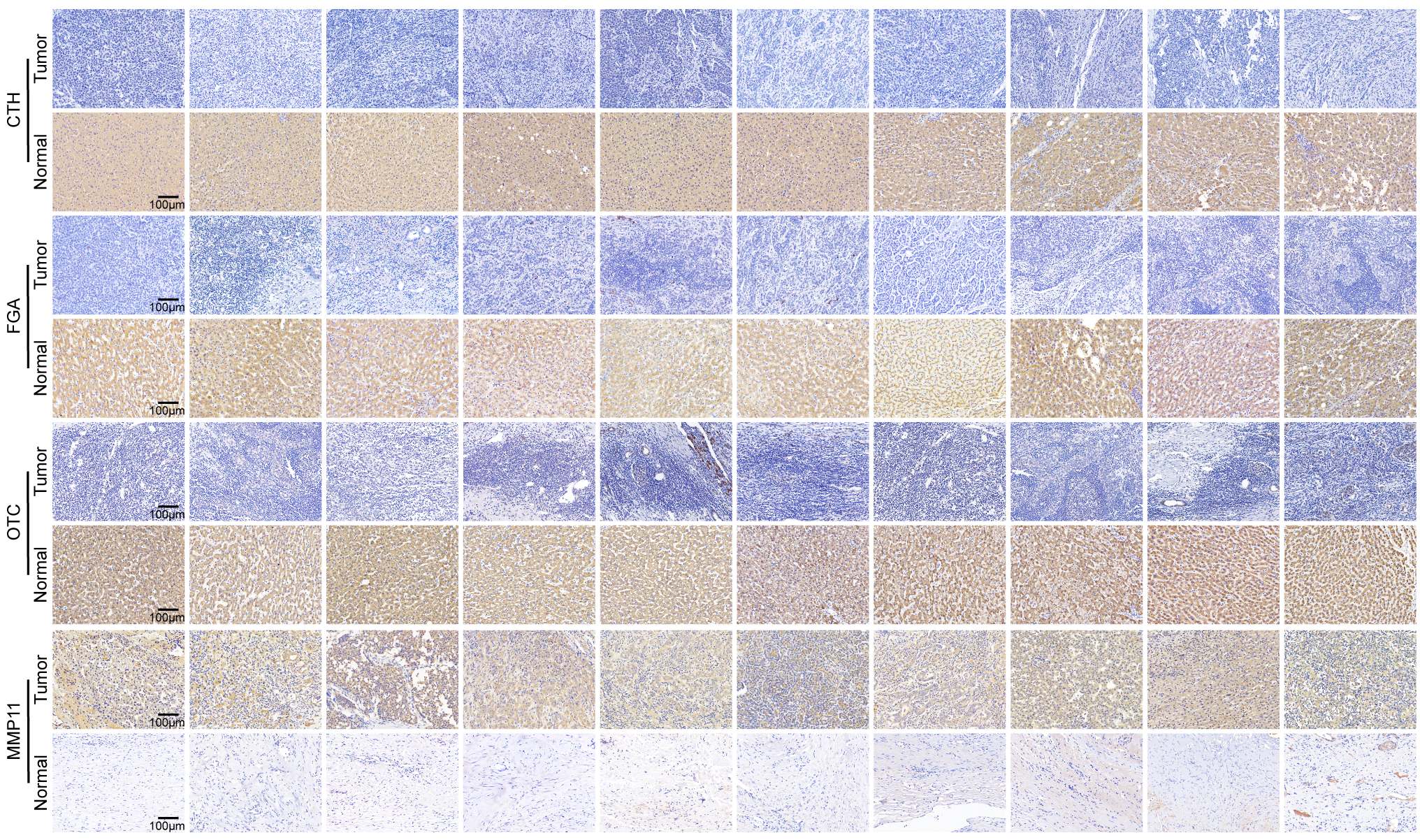

Supplement: Supplementary Figure 2 — Immunohistochemical results of CTH, FGA, OTC and MMP11 of 10 CCA samples other than Figure 7 . [file Image_2.tif]
