# Supplementary material for: Screening potential biomarkers of cholangiocarcinoma based on gene chip meta-analysis and small-sample experimental research
Source: Front Oncol. 2022 Oct 10;12:1001400. doi: 10.3389/fonc.2022.1001400 (PMC9590411; doi:10.3389/fonc.2022.1001400)
Supplement: Supplementary file 3 [file Table_1.docx]

**Supplementary Table 1.** DEGs correlation with 10 biosets curated in BSCE (-Log(p-values)

| **Bioset name** | **GSE26566-1** | **GSE31370-1** | **GSE26566-2** | **GSE15765-1** | **GSE15765-2** | **GSE32225-2** | **GSE31370-2** | **GSE32225-1** | **GSE77984** | **GSE34166** |
| --- | --- | --- | --- | --- | --- | --- | --- | --- | --- | --- |
| **ADH1A_GSE26566-1** | 0 | 272 | 220 | 100 | 48 | 25 | 137 | 45 | 22 | 12 |
| **ALDOB_GSE31370-1** | 272 | 0 | 127 | 176 | 67 | 44 | 0 | 77 | 25 | 15 |
| **AOX1_GSE26566-2** | 220 | 127 | 0 | 57 | 30 | 20 | 80 | 40 | 34 | 7 |
| **CEACAM6_GSE77984** | 22 | 25 | 34 | 26 | 2 | 5 | 6 | 7 | 0 | 1 |
| **CTH_GSE15765-1** | 100 | 176 | 57 | 0 | 190 | 18 | 70 | 35 | 26 | 30 |
| **EPCAM_GSE31370-2** | 272 | 0 | 127 | 176 | 67 | 44 | 0 | 77 | 25 | 15 |
| **FGA_GSE32225-2** | 25 | 44 | 20 | 18 | 11 | 0 | 25 | 163 | 5 | 2 |
| **FGB_GSE31370-2** | 137 | 0 | 80 | 70 | 31 | 25 | 0 | 54 | 6 | 12 |
| **FGG_GSE32225-1** | 45 | 77 | 40 | 35 | 22 | 163 | 54 | 0 | 7 | 4 |
| **GSTA1_GSE34166** | 12 | 15 | 7 | 30 | 9 | 2 | 12 | 4 | 1 | 0 |
| **KRT17_GSE32225-2** | 25 | 44 | 20 | 18 | 11 | 0 | 25 | 163 | 5 | 2 |
| **KRT19_GSE15765-2** | 48 | 67 | 30 | 190 | 0 | 11 | 31 | 22 | 2 | 9 |
| **KRT7_GSE31370-1** | 137 | 0 | 80 | 70 | 31 | 25 | 0 | 54 | 6 | 12 |
| **LAMC2_GSE15765-1** | 100 | 176 | 57 | 0 | 190 | 18 | 70 | 35 | 26 | 30 |
| **MMP11_GSE15765-1** | 100 | 176 | 57 | 0 | 190 | 18 | 70 | 35 | 26 | 30 |
| **OTC_GSE26566-1** | 0 | 272 | 220 | 100 | 48 | 25 | 137 | 45 | 22 | 12 |
| **SFN_GSE32225-1** | 45 | 77 | 40 | 35 | 22 | 163 | 54 | 0 | 7 | 4 |
| **SOX9_GSE15765-2** | 48 | 67 | 30 | 190 | 0 | 11 | 31 | 22 | 2 | 9 |
